# Primary and Revision Reverse Shoulder Arthroplasty Using Custom-Made 3D-Printed Baseplates for Severe Multiplanar Glenoid Bone Defects: A Retrospective Study of Clinical and Radiographic Outcomes

**DOI:** 10.3390/jcm14176153

**Published:** 2025-08-30

**Authors:** Giovanni Merolla, Francesco De Filippo, Fabiana Magrini Pasquinelli, Gian Mario Micheloni, Giuseppe Porcellini, Paolo Paladini, Roberto Castricini

**Affiliations:** 1 Shoulder and Elbow Unit, Cervesi Hospital, AUSL Romagna, 47841 Cattolica, Italy; df.francesco28@virgilio.it (F.D.F.); palpaolo@tin.it (P.P.); 2 Center of Upper Limb Arthroscopy, Trauma and Minimally Invasive Surgery, Nova Clinic, 47895 Domagnano, San Marino; 3 Orthopaedic and Trauma Unit, Principe di Piemonte Hospital, 60019 Senigallia, Italy; fabiana.magrini@gmail.com; 4 Orthopedic and Trauma Unit, Sassuolo Hospital, University of Modena and Reggio Emilia, 41049 Sassuolo, Italy; gianmario.micheloni@gmail.com (G.M.M.); prof.giuseppeporcellini@gmail.com (G.P.); 5 Division of Orthopaedic and Trauma Surgery, Villa Verde Hospital, 63900 Fermo, Italy; robertocastricini@gmail.com

**Keywords:** reverse shoulder arthroplasty, glenoid bone loss, revision, anatomical models, custom implant

## Abstract

**Background:** Severe glenoid bone loss presents a major challenge in both primary and revision reverse shoulder arthroplasty (RSA). Standard implants often fail to achieve reliable fixation in these cases. Custom-made, 3D-printed glenoid components have emerged as a potential solution, offering anatomically tailored fit and fixation. This study evaluates the clinical and radiographic outcomes of custom-made glenoid implants in managing severe glenoid bone loss. **Methods:** A retrospective, multicenter study was conducted on 23 shoulders (11 primary and 12 revision RSAs) that received a custom-made glenoid component using the Enovis ProMade System (San Daniele del Friuli, Udine, Italy) between 2017 and 2022, with a minimum follow-up of 24 months. Preoperative planning utilized CT-based 3D modeling to design implants with patient-specific instrumentation. Clinical outcomes (ROM, pain, Constant–Murley score) and radiographic results were assessed. Statistical comparisons were made between primary and revision groups. **Results:** Both groups demonstrated significant improvements in shoulder mobility, pain relief, and Constant–Murley scores (all *p* < 0.001), with no significant differences between primary and revision groups in delta scores. Radiographically, no loosening was observed, with minimal radiolucent lines and low complication rates. Four cases of instability occurred, all in the revision group, with only one requiring conversion to hemiarthroplasty. No differences in radiographic outcomes were observed between groups. **Conclusions:** Custom-made glenoid implants provide a reliable solution for severe glenoid bone loss in both primary and revision RSA, yielding consistent functional improvement and implant stability. Further prospective studies with larger cohorts and long-term follow-up are warranted to confirm these findings and assess cost-effectiveness.

## 1. Introduction

The increased number of primary shoulder arthroplasties and the consequent rise in revision procedures, made the management of glenoid bone defects a major challenge in contemporary shoulder surgery [1,2,3]. Glenoid bone defects may result from a range of etiologies, including degenerative joint disease, congenital abnormalities, recurrent instability, autoimmune disorders, complications related to fracture management, or as a consequence of primary or revision shoulder arthroplasty [4].

Although several classification systems have been proposed for severe glenoid bone defects, the quantitative assessment and optimal treatment of glenoid bone loss remain subjects of ongoing debate [5,6].

Several surgical techniques have been described to address glenoid bone loss. In cases of primary osteoarthritis with mild-to-moderate defects, such as Walch B2 or B3 glenoid morphology [7], standard total shoulder arthroplasty with eccentric glenoid reaming, bony increased-offset reverse shoulder arthroplasty (BIO-RSA), and metal increased-offset reverse shoulder arthroplasty (MIO-RSA) have demonstrated favorable mid-term clinical outcomes [8,9].

However, the management of severe central and peripheral glenoid defects remains a significant concern, particularly in revision shoulder arthroplasty. In these cases, shoulder hemiarthroplasty combined with glenoid bone grafting has produced inconsistent outcomes, with a notable incidence of persistent postoperative shoulder pain [10,11,12,13,14].

With the advancement of 3D CT imaging techniques and their application in the development of custom-made prostheses, it is now possible to design glenoid components that are precisely tailored to the native bone defect [15]. This represents a promising solution for cases of severe glenoid bone loss, where standard implants often fail to provide reliable fixation and stability.

Although current evidence in the literature is still limited, custom-made glenoid components have shown the potential to deliver good functional outcomes, stable implant fixation, and low complication rates in patients with substantial glenoid bone loss [16,17,18,19,20].

The aim of this study was to evaluate the clinical and radiographic outcomes of 3D-printed custom-made glenoid implants in both primary and revision shoulder arthroplasty involving severe glenoid bone loss.

## 2. Materials and Methods

### 2.1. Study Design

This was a retrospective analysis of data collected from January 2017 to December 2022 in four orthopedic high-volume units and was approved by the institutional review board of coordinator center as part of a larger Shoulder Arthroplasty investigation (prot. n. 6478/2019 I.5/117).

All patients who underwent RSA with implantation of a 3D-printed glenoid component using the Enovis ProMade System (Enovis, San Daniele del Friuli, Udine, Italy) and a minimum follow-up of 24 months were included. Both primary and revision procedures were considered in the presence of a severe glenoid bone defect that precluded the use of a standard primary or revision implant. There were no specific exclusion criteria.

All data were collected through the review of operative reports, pre- and postoperative imaging, and hospital discharge summaries. In addition, all patients included in the study underwent a clinical and radiographic evaluation at a minimum follow-up of 24 months following the surgical procedure.

Overall, 23 patients (23 shoulders) with complete clinical and radiographic data were included.

We identified patients who received primary implants (primary group, 11 shoulders) and those who underwent revision procedures (revision group, 12 shoulders).

The demographics and perioperative data of the two groups are reported in Table 1.

### 2.2. Preoperative Imaging

Preoperative imaging included plain radiographs (anterior–posterior Grashey, Y lateral, and axillary views) and computed tomography (CT) scans.

Established criteria were used to grade arthritis severity and the type of glenoid erosion [5,7,21].

Fatty infiltration was graded according to Goutallier et al. [22].

Arthritis grade and glenoid type are reported in Table 1.

### 2.3. Implant Design and Custom-Made Development Process

**Figure 1:**
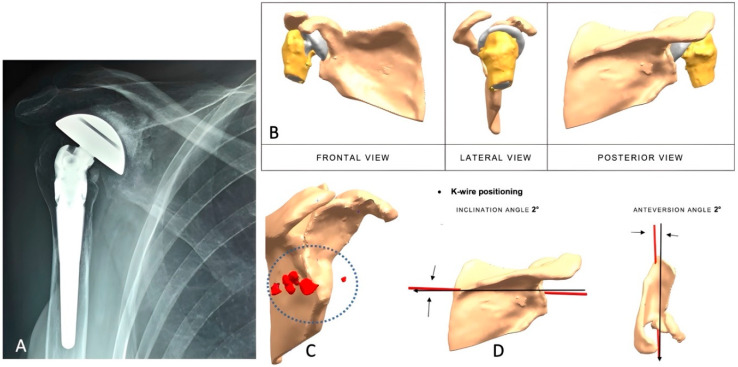
(**A**–**D**): Painful reverse shoulder arthroplasty in a 56-year-old female patient with a history of multiple surgeries. (**A**) Antero-posterior radiograph showing superior migration of the humeral component and severe glenoid erosion. (**B**) CT scan reconstruction with digital removal of metallic artifacts from previous implant. (**C**) Scapular reconstruction revealing a multiplanar glenoid defect and multiple bone fragments on the posterior aspect of the glenoid (circle with red items). (**D**) Orientation of the K-wire guide showing inclination and anteversion for central peg placement.

**Figure 2:**
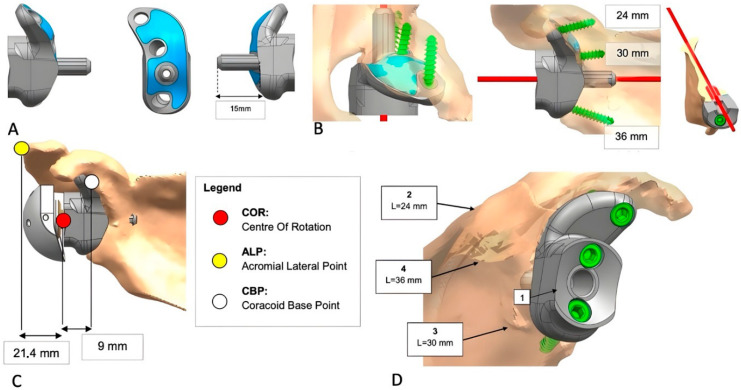
(**A**–**D**): Depiction of implant features and their adaptation to the glenoid bone defect. (**A**) Implant dimensions and characteristics; the blue color indicates TT^®^ coating. (**B**) Representation of the position and length of the peripheral screws and central peg. (**C**) Implant lateralization achieved using a 44 mm glenosphere. (**D**) Final implant configuration (1: baseplate; 2–4: lengths of screws).

A biomedical engineer reconstructed the scapula using CT-based segmentation to generate a 3D model for quantitative assessment of bone stock and glenoid defects. In revision cases, metallic artifacts were digitally removed. The custom implant was then designed to conform to the bone defect, optimizing baseplate fixation. Biomechanical and finite element analyses evaluated joint mechanics and implant performance under physiological loading. Patient-specific instruments (PSIs) and surgical steps were developed to support accurate intraoperative placement. Upon surgeon approval, implants were 3D-printed in Trabecular Titanium^®^ and machined at critical interfaces. PSIs and anatomical models were printed in polyamide, with lateralization assessed using the center of rotation (COR), acromial lateral point (ALP), and coracoid base point (CBP) as benchmarks (Figure 1A–D and Figure 2A–D).

### 2.4. Clinical Evaluation and Outcome Measures

Clinical status was assessed before the procedure and at the last follow-up. Active range of motion (ROM), pain (with a visual analog scale, VAS) and the Constant–Murley scores (ref) were evaluated at the same time points. Active ROM was assessed in terms of anterior elevation (AAE), lateral elevation (ALE), external rotation (ER) with the patient standing using a goniometer, and internal rotation (IR) as the ability to reach different levels of the spine with the thumb. Pain was graded from 0 to 10, where 0 was no pain. ROM and clinical scores were assessed by an examiner who did not take part in the surgical procedures.

### 2.5. Operative Procedure

All procedures were performed in the beach-chair position under general anesthesia combined with regional nerve block. A standard deltopectoral approach was used. If intact, the subscapularis was detached via tenotomy, and the humerus was dislocated. The humeral head was resected at the anatomical neck, followed by glenoid exposure.

Extensive debridement of fibrotic tissue and non-contributory bony prominences was performed to achieve a surface that matched the preoperative 3D planning. Particular attention was given to exposing the base of the coracoid process, a critical anatomical landmark for correct positioning of the patient-specific implant and surgical guides (PSIs).

The PSI was first trialed on a 3D-printed glenoid model, then positioned intraoperatively to guide central guidewire insertion. The glenoid peg socket was prepared using dedicated instrumentation for accurate depth and orientation. The custom-made glenoid component was impacted, and, once proper seating was confirmed, fixation screws and the glenosphere were implanted using dedicated guides.

The humerus was hand-reamed to achieve a press-fit, and joint stability was assessed with trial components. Once satisfactory tension and range of motion were confirmed, the definitive humeral component was implanted. Patients with significant proximal humeral bone loss received long stems (150 or 180 mm) without glenoid augmentation. The subscapularis was reattached when possible, and the wound was closed in layers. Postoperatively, the arm was immobilized in a sling for 3 weeks.

### 2.6. Postoperative Imaging

Radiographs were obtained in the immediate postoperative period and at the last follow-up evaluation for the need of the current study. Radiological changes around the reverse implants were assessed using previously reported criteria for the humeral component (radiolucency, condensation lines, cortical thinning, spot weld, subsidence and resorption of the tuberosities, and loosening) and the glenoid component (radiolucency, scapular notching, formation of bony scapular spurs, ossifications, and loosening) [21,23]. Humeral radiolucent lines were assessed in 7 areas. A blinded observer, who did not take part in the surgical procedures, reviewed the radiograms and also assessed any periscapular fractures defined according to Levy et al. [24].

### 2.7. Statistical Analysis

Descriptive statistics (absolute and percent frequency, mean, median, standard deviation [SD], and range) for each group were calculated for all variables. Delta scores were calculated for clinical scores as the difference between postoperative and preoperative values. The preoperative scores and delta scores of the two groups were compared using the Mann–Whitney test.

The possible association between each group and the qualitative variables, either baseline and postoperative, were analyzed with Pearson’s χ^2^ test.

The level of significance was set at 0.05. All analyses were performed with Stata Intercooled 9.2 software for Windows.

## 3. Results

There were no differences in size, age, gender, or rotator cuff fatty infiltration between the groups. Mean follow-up duration was 24.1 months (SD: 7.7) in the primary group and 32.2 months (SD: 15.3) in the revision group (Table 1).

### 3.1. Clinical Outcomes

The preoperative and postoperative delta scores for AAE, ALE, ER, and IR showed significant differences in both groups (all *p* < 0.0001). Pain and CS also improved significantly: pain decreased by a median of 8 points in the revision group and 7 points in the primary group (*p* < 0.001). The CS increased by a median of 24 points in the primary group and 34 points in the revision group (all *p* < 0.0001).

No significant correlation was found between fatty infiltration of the posterior rotator cuff muscles (i.e., infraspinatus and teres minor) and improvement in ER. There were no significant differences in the delta scores for shoulder mobility, pain, or CS between the primary and revision groups (Table 2 and Table 3).

However, the primary arthroplasty group showed higher DLA and mobility sub-scores of the CS (*p* = 0.04 and *p* = 0.03, respectively). Glenosphere eccentricity, size, and tilt had no significant effect on clinical scores or shoulder mobility.

### 3.2. Radiographic Outcomes

Glenoid bone defects in the two group were classified as shown in Table 1. The mean global glenoid offset recorded on anatomical models was 29.8 mm. No cases demonstrating significant proximal humeral bone loss were identified.

We found one shoulder with grade I scapular notching and HO; RL < 2 mm was found around the glenoid component in six shoulders (25%) and around the humeral component in four shoulders (17%). Humeral cortical thinning was depicted in four cases (17%).

A subgroup analysis showed no differences in radiographic outcomes between the primary and revision group.

### 3.3. Complications and Revisions

**Figure 3:**
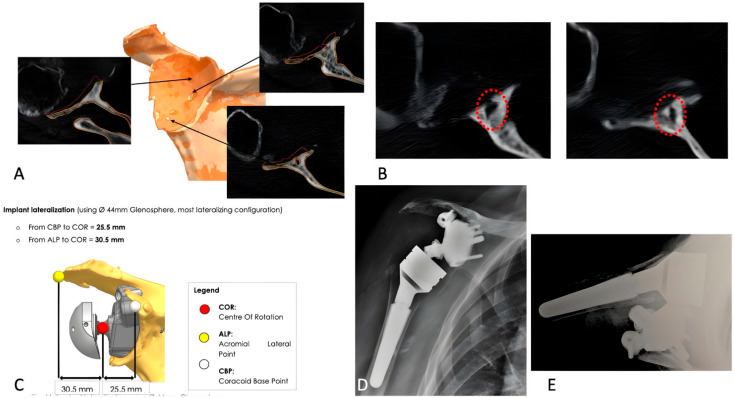
(**A**–**E**): Case description from the primary group. (**A**) 2D and 3D CT scans showing a severe central glenoid defect with thinning of the anterior and posterior glenoid borders. (**B**) Degenerative cyst in the central glenoid causing osteolysis (red circles). (**C**) Anatomical reconstruction with baseplate fixation and lateralization using a 44 mm glenosphere. (**D**) Postoperative anteroposterior radiograph. (**E**) Axillary view showing anterior dislocation of the humerus three months postoperatively.

**Figure 4:**
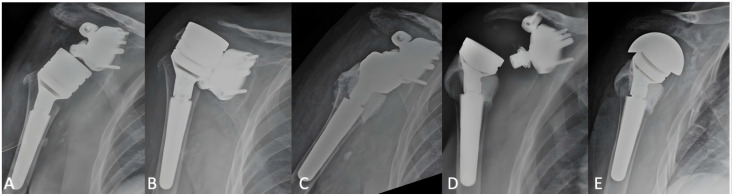
(**A**–**E**): Postoperative radiographs of the unstable reverse shoulder arthroplasty (RSA) described in Figure 3. (**A**) Anteroposterior view following revision with a metallic humeral spacer. (**B**) Dislocation observed two months after the revision. (**C**) Second revision procedure involving removal of the metallic spacer and cement filling of the proximal humeral metaphysis to increase the deltoid wrapping angle. (**D**) Dislocation noted three months after the second revision. (**E**) Final revision with conversion to hemiarthroplasty.

Four cases of RSA instability (17%) were observed. The first patient experienced recurrent anterior-superior dislocations despite two revisions, including spacer insertion and humeral component cementation, and was ultimately converted to hemiarthroplasty (see Figure 3A–E and Figure 4A–E). The second case involved anterior dislocation at 2 months; revision with larger components led to stable, pain-free function. The third case had dislocation at 75 days, which spontaneously reduced; the patient declined revision and is undergoing rehabilitation. The fourth patient had a posterior dislocation at 4 months, managed with closed reduction. However, a humeral metaphyseal fracture was later identified and treated conservatively. The implant remains stable, and the patient is pain-free.

## 4. Discussion

The management of severe glenoid bone loss remains a significant challenge in both primary and revision RSA. Despite advancements in implant design and surgical techniques, the optimal approach for addressing these complex cases continues to be debated.

The main finding of this study was the effectiveness of custom-made glenoid implants in improving pain and functional outcomes in both primary and revision RSA, with no significant differences observed between the two groups. The comparable results in clinical outcomes—such as range of motion, Constant score, and pain relief—suggest that patient-specific implants can mitigate the anatomical challenges typically associated with revision procedures. Although outcomes in revision cases may not fully match those of primary RSA, they can still be considered clinically satisfactory.

However, the similarity in postoperative outcomes should be interpreted with caution due to the limited sample size and wide confidence intervals, indicating substantial variability in pre- and postoperative scores. Additionally, several confounding factors—particularly in revision cases—must be considered, including the number of prior surgeries, glenoid defect type, extent of humeral bone loss, rotator cuff integrity, and patient comorbidities. These factors can significantly impact clinical results and complication rates, thereby limiting the reliability of direct comparisons between primary and revision procedures.

These results are consistent with those reported by Rashid et al. [20], who found comparable outcomes between primary and revision procedures using the same custom-made glenoid implants. Similarly, Ortmaier et al. [18] documented a mean improvement of 31 points in Constant score among revision cases, while Michelin et al. [25] reported mean Constant score gains of 33 points in primary procedures and 27 points in revisions. These findings closely mirror our own results, with mean improvements of 35 points in the primary group and 25 points in the revision group. Although no significant differences were observed in the overall delta Constant scores between the primary and revision groups, some subscores—specifically activities of daily living and mobility—demonstrated slightly better outcomes in the primary group. This trend may reflect the generally more favorable soft tissue quality typically encountered in primary procedures compared to revision settings [17,26]. Addressing soft tissue deficiencies of the shoulder following multiple surgeries and severe glenoid bone loss remains a significant concern. The use of cement or structural bone grafts in shoulders with proximal humeral bone loss has been advocated to improve the deltoid “wrapping angle,” thereby enhancing joint stability [27]. Additionally, lateralization of the glenosphere has been proposed to optimize deltoid tension and its coaptation force [28,29]. In our experience, inadequate soft tissue tension and muscular imbalance represent major challenges in revision shoulder arthroplasty. These factors significantly increase the risk of implant instability, which can be difficult to manage or correct. A definitive solution to this issue in reverse shoulder arthroplasty (RSA) remains elusive, as illustrated by one case in our series that required conversion to hemiarthroplasty following multiple failed revision attempts.

In this study, lateralization of approximately 3 cm—planned using preoperative anatomical modeling—may still be insufficient in patients with poor deltoid tension and strength. Our objective was to optimize RSA distalization in combination with appropriate glenoid lateralization, aiming to enhance shoulder function and reduce pain, consistent with the biomechanical principles of Grammont-style RSA [30]. The precise impact of distalization and lateralization on joint function in newer reverse shoulder arthroplasty (RSA) designs remains uncertain [31,32].

Four cases of instability were observed in our series, resulting in an overall complication rate of 17%. These cases warrant further consideration.

We believe the surgical approach can be excluded as a contributing factor, as all procedures were performed by surgeons with comparable training and experience in shoulder surgery. Notably, all instability cases occurred within the revision group, reinforcing concerns regarding the impact of multiple prior surgeries on muscle tension and load distribution. These findings support the previously discussed considerations related to soft tissue quality and deltoid wrapping.

Among these cases, the one requiring conversion to HA may have played a key role in the persistence of instability. In contrast, modification of the components to increase lateralization in another case led to improved joint stability, with a beneficial effect on implant fixation [28,29,32].

In one patient, poor bone quality resulted in a humeral metaphyseal fracture, providing a mechanical explanation for the observed instability.

No conclusions can be drawn regarding the remaining case, as the patient declined revision surgery, limiting further evaluation.

This complication rate is consistent with previously reported data. Rashid et al. [20] observed a 9.3% complication rate, while Michelin et al. [25] and Debeer et al. [33] reported higher rates of 29.1% and 20%, respectively. In contrast, both Bodendorfer et al. [17] and Ortmaier et al. [18] reported no complications in their respective series, though their sample sizes were limited. This finding suggests that custom-made implants provide reliable performance over time in both primary and revision settings, as previously demonstrated by other authors who reported stable functional and radiological outcomes at mid-to-long-term follow-up [16,20,25]. Although the revision group in our cohort had a longer mean follow-up period, this was not associated with any significant differences in clinical outcomes.

Compared to alternative approaches such as structural bone grafting, custom-made implants appear to be associated with lower rates of graft-related complications, including resorption, non-union, and eventual glenoid loosening.

In a recent study involving 26 patients undergoing glenoid reconstruction with structural bone grafts, Sholtis et al. reported a complication rate of 27% and a revision rate of 19% [34].

Custom-made implants may help reduce complications related to graft integration by achieving mechanical stability through a precise anatomic fit and optimized screw fixation points, thereby bypassing the need for biological incorporation of the graft.

The cost-effectiveness of customized glenoid reverse shoulder arthroplasty using 3D-printed, patient-specific implants remains under investigation. Although these custom implants offer potential advantages—such as enhanced implant positioning and improved bone stock preservation—they are associated with higher upfront costs and necessitate specialized surgical expertise. Notably, Yam et al. (2021) [35] reported a significant cost reduction when utilizing in-house 3D-printed surgical jigs for RSA compared to those from commercial vendors, a strategy that our unit is similarly adopting.

A 3D-printed guiding baseplate also represents a viable option for facilitating accurate glenoid prosthesis implantation in the context of orthopedic oncology. RSA utilizing a 3D-printed glenoid component in combination with a personalized, custom-made humeral prosthesis has been shown to improve shoulder function and reduce complication rates [36,37]. While the manufacturing process for 3D-printed implants remains time-consuming and may not be suitable for emergency cases, we are optimistic that advancements in digital technology will soon enable more rapid and widespread use of 3D printing, even in urgent oncologic settings.

### Conclusions and Limitations of the Study

In conclusion, this study supports the use of custom-made glenoid implants as an effective solution for managing severe glenoid bone loss, both in primary and revision reverse shoulder arthroplasty.

Nonetheless, the retrospective nature of the study, the relatively small sample size, and the limited follow-up represent important limitations.

We acknowledge that small sample sizes can result in statistically fragile findings, potentially overestimating clinical outcomes, misrepresenting true complication rates, or limiting the ability to draw reliable conclusions. However, it is important to note that custom glenoid implants represent a rare subset of RSA. Indeed, a multicenter study was necessary to obtain a sufficient number of cases for meaningful analysis.

Further prospective studies with larger cohorts, longer follow-up, and direct comparisons with standard reconstruction techniques, matched for complexity, are needed to validate these findings. In addition, cost-effectiveness analyses would be valuable to assess the clinical impact of custom-made solutions.

## Figures and Tables

**Table 1:** Demographic and perioperative data of the 2 groups of patients.

Variable	Primary RSA	Revision RSA	*p* Value
Shoulders (no.)	11	12	0.429
Follow-up duration, mo, median (range)	24.09 (7.7)	32.25 (15.29)	0.174
Mean age (years) (range)	66.45 (40–80)	67.58 (48–86)	0.82
Gender (M/F) (%)	4/7	7/5	0.52
Glenoid wear according to Sirveaux et al. [21] (no.) (%)			NA
E0	0	0	
E1	1 (9.1)	0	
E2	0	0	
E3	5 (45.5)	1 (8.3)	
Glenoid bone loss according to Bercik-Walch et al. [7] (no.) (%)			NA
A1	0	0	
A2	0	0	
B1	0	0	
B2	0	0	
B3	0	0	
C	0	1 (8.3)	
Glenoid bone loss according to Gupta-Seebauer [5] (no.) (%)			0.565
C1	0	0	
C2	0	0	
C3	1 (9.1)	0	
C4	2 (18.2)	4 (33.3)	
E1	0	0	
E2	0	0	
E3	1 (9.1)	1 (8.3)	
E4	3 (27.3)	4 (33.3)	
Cemented stem	1 (9.1)	1 (8.3)	

NA: not applicable.

**Table 2:** Active shoulder mobility in primary and revision group.

	Primary Arthroplasty Group			Revision Arthroplasty Group			
Variable	Median (IQR)	Mean ± SD	Confidence Interval	Median (IQR)	Mean ± SD	Confidence Interval	*p* Value (Mann–Witney Test)
AAE_1_	70 (60; 90)	71.8 ± 20.6		70 (40; 70)	59.6 ± 17.4		0.19
AAE_2_	125 (105; 130)	118.6 ± 14		105 (65; 126)	99.3 ± 41.1		0.18
AAE Delta score	−55 (−65; −35)	−46.8 ± 30.1	−67.0; −26.6	−31 (−77.5; −10)	−39.7 ± 35.3	−62.2; −17.3	0.57
ALE_1_	50 (45; 70)	52.7 ± 16.6		45 (10; 60)	39.2 ± 26.7		0.24
ALE_2_	100 (100; 110)	101.4 ± 11.4		82.5 (65; 100)	80.4 ± 4.2		0.07
ALE Delta score	−50 (−60; −40)	−48.6 ± 16.3	−59.6; −37.7	−32.5 (−57.5; −5)	−41.2 ± 44.9	−69.8; −12.8	0.17
ER_1_	2 (2; 2)	1.6 ± 0.8		2 (2; 2)	1.7 ± 0.78		0.92
ER_2_	2 (2; 6)	3.3 ± 1.8		2 (2; 2)	2 ± 0.85		0.06
ER delta score	0 (+4; 0)	−1.6 ± 2	−3.0; −0.32	0 (0; 0)	−0.33 ± 1.4	−1.2; −0.58	0.08
IR_1_	2 (2; 4)	2.5 ± 1.3		1 (0; 2)	1.3 ± 1.6		0.06
IR_2_	4 (4; 4)	4.5 ± 1.3		4 (1; 6)	3.7 ± 2.9		0.40
IR delta score	−2 (−4; 0)	−2 ± 1.5	−3.0; −0.96	−3 (−4; 0)	−2.3 ± 3.4	−4.5; −0.18	0.61

IQR: interquartile range (25th–75th percentile); SD: standard deviation; AAE: active forward elevation; ALE: active lateral elevation; ER: external rotation; IR: internal rotation; AAE and ER are reported in degree; IR is reported in points: 0 = dorsum of hand to lateral thigh and 10 = dorsum of hand to interscapular region. Subscripts 1 and 2 indicate preoperative and postoperative values, respectively; Delta scores: difference between preoperative and postoperative values. The difference between the delta scores of the two groups at the last follow-up evaluation were analyzed with the Mann–Whitney test.

**Table 3:** Clinical scores in primary and revision group.

	Primary Arthroplasty Group			Revision Arthroplasty Group			
Variable	Median (IQR)	Mean ± SD	Confidence Interval	Median (IQR)	Mean ± SD	Confidence Interval	*p* Value (Mann–Whitney)
CS_1_	22 (20; 30)	23.3 ± 6.6		20 (12; 24)	19.5 ± 8.7		0.17
CS_2_	58 (55; 64)	58.8 ± 4.2		46 (29; 61)	45.2 ± 20.2		0.09
CS_2pain_	12 (10; 5)	12.4 ± 2.2		10 (10; 15)	10.8 ± 3.6		0.23
CS_2DLA_	16 (14; 20)	15.9 ± 4.2		13 (7; 16)	11.7 ± 5.1		0.04
CS_2mobility_	22 (20; 26)	24.2 ± 7.1		14.5 (11; 22)	16.2 ± 8.7		0.03
CS_2strength_	4 (2; 8)	4.7 ± 2.4		4 (1.5; 5)	4.5 ± 4.2		0.39
CS delta score	−34 (−37; −28)	−35.5 ± 8.7	−41.39;−29.7	−24 (−37.5; −10.5)	−25.7 ± 16	−35.9; −15.6	0.09

IQR: interquartile range (25th–75th percentile); SD: standard deviation; CS: Constant–Murley score; The subscript 1 and 2 numbers indicate preoperative and postoperative values, respectively; DLA: daily living activity.

## Data Availability

The original contributions presented in this study are included in the article. Further inquiries can be directed to the corresponding author.
